# Influence of twin-headed gemini micellar system on the study of methionine amino acid with ninhydrin in buffer solution

**DOI:** 10.1098/rsos.221249

**Published:** 2023-02-15

**Authors:** Yousef G. Alghamdi, Malik Abdul Rub, Dileep Kumar

**Affiliations:** ^1^ Chemistry Department, Faculty of Science, King Abdulaziz University, Jeddah-21589, Saudi Arabia; ^2^ Center of Excellence for Advanced Materials Research, King Abdulaziz University, Jeddah-21589, Saudi Arabia; ^3^ Laboratory for Chemical Computation and Modeling, Institute for Computational Science and Artificial Intelligence, Van Lang University, Ho Chi Minh City, Vietnam; ^4^ Faculty of Applied Technology, School of Technology, Van Lang University, Ho Chi Minh City, Vietnam

**Keywords:** conductivity measurements, double-headed geminis, interfacial activity, methionine, spectrophotometer

## Abstract

The influence of double-headed gemini was examined in the present study by studying the amino acid methionine mixed with ninhydrin in CH_3_COOH-CH_3_COONa buffer solvent. The absorbance was monitored at fixed time intervals with UV-vis spectroscopy. An impact typical of surfactants was observed on the ninhydrin–methionine reaction and explained by a pseudo-phase model of micelles. The effect of different temperatures (343 to 363 K) was also determined. Based on data showing the impact of temperature on *k_ψ_*, several relevant thermodynamic quantities, Δ*H*^#^, Δ*S*^#^, and *E*_a_, were calculated using linear least-squares regression. In addition, the influence of the other reaction ingredients on the reaction, that is, pH and the concentration of ninhydrin and methionine, was studied. The CMC (critical micelle concentration) of pure geminis and the surfactant system with methionine and ninhydrin was evaluated at two temperatures, i.e. at 303 K and 353 K by conductivity measurements. The CMC values of pure gemini surfactants evaluated in the existing case at 303 K are concordant with the results stated before. Moreover, other parameters, including rates and binding constants, were calculated.

## 1. Introduction

A surfactant is an amphiphilic molecule with two distinct segments: a hydrophilic head and a hydrocarbon tail. Generally, surfactant molecules can be characterized into four categories: nonionic, anionic, cationic and amphipathic. Their nature depends on the charge of the hydrophilic head group [1–8]. Because of the dual nature of a surfactant system, they differ from other surface-active materials. Owing to this special molecular architecture, surfactants lower the high interfacial tension through adsorption at the air–water interface.

Today, surfactants have numerous uses in various applications, including chemicals used daily, micellar catalysis, pharmaceutics, drug delivery, paintings and corrosion treatments. [8–15]. These applications require a better understanding and evaluation of the critical micelle concentration (CMC). Surfactants associate at small concentrations, denoted the CMC. The value of CMC can be measured at the sudden change of any physical properties over a small range of surfactant concentrations. It is a particularly advantageous parameter because it quantifies the surface and interfacial behaviour of surfactants in aqueous solution. In order to determine the CMC, several tools are used, including tensiometry, UV-vis spectroscopy, conductivity, fluorometry and densitometry [16–22].

An outstanding surfactant, gemini, has numerous applications, including the food and textile industries, recovery of tertiary oil and medical science, due to its interfacial properties [23–27]. However, the complex synthesis and purification process of this surfactant impedes its use in different industrial applications. Gemini surfactants comprise two surfactant monomers connected at the head position through a linker. These surfactants have received academic and industrial attention due to their excellent properties (e.g. advanced wettability, smaller CMC, superior solubilizing ability) compared to single-chain surfactants [28–36]. The nature of the hydrophilic heads and the linker of gemini surfactants impacts their use through several physico-chemical phenomena. As a consequence, their molecular arrangement and exceptional properties make geminis valuable for applications at a large scale in various sectors.

Due to different potential advanced synthetic routes and structural properties, researchers are paying more attention to gemini surfactants. Their unusual and enhanced behaviour is also of interest to scientists working in surface and interface sciences. Many papers have been published regarding the impact of gemini surfactants, the morphologies they form on surfaces, and the interfacial phenomena studied using various experimental instruments, including UV-vis spectrophotometer, fluorometer, conductivity meter, NMR spectrometer, DLS and tensiometer [37–44]. Although large studies have been performed on the surface behaviour of geminis, the influence of double-headed gemini on the reaction rate in buffer solutions has not been reported very often, and satisfactory results have not been obtained. Further studies are required for better understanding and improved function.

Ninhydrin-concerning reactions are applied frequently to analyze amine functionality in several fields (forensics, agriculture, biochemical analysis and biomedical work). It is a unique and superior colour-producing compound. The interaction of ninhydrin with amino groups in a buffer solvent produces a coloured compound (diketohydrindylidene–diketohydrindamine, DYDA or Ruhemann's purple) [45–47]. The colour of DYDA fades at room temperature and becomes unstable. Many works to improve the stability of this compound have been conducted by studying the effects of surfactant monomers (CPB and CTAB), several aqueous and non-aqueous solvents, and various salts. In these studies, the product formed was enhanced and stable for longer times [48–53]. However, the influence of double-headed gemini on amine functionality with ninhydrin in buffer solution has been reported in a very limited way and has not been specifically studied.

We have synthesized double-headed gemini surfactants (16-6-16, GS-6; 16-5-16, GS-5 and 16-4-16, GS-4) in the present study. The impact of these synthesized geminis on the interaction of methionine with ninhydrin in a buffer solvent was carefully examined. This work shows that GS-6, GS-5 and GS-4 geminis may be categorized as more beneficial surfactants (inexpensive and environmentally acceptable). The results obtained in the present case are compared with previous studies.

## 2. Experimental section

### 2.1. Material and methods

Double-distilled water was used to prepare solutions. Sodium acetate–acetic acid buffer was used as a solvent throughout the study; it was prepared with 0.2 mol dm^−3^ of 30 cm^3^ CH_3_COOH and 0.2 mol dm^−3^ of 70 cm^3^ CH_3_COONa solutions. Relevant details regarding the preparation of buffer solutions are provided elsewhere [54]. All materials used were of pure analytical grade and employed as supplied. The synthesizing materials, N, N-dimethylcetylamine (95%), 1,6-dibromohexane (97%), 1,4-dibromobutane (98%), and 1,5-dibromopentane (98%) were bought from Fluka, Germany. The chemicals 2,2-dihydroxy-1,3-indanedione (ninhydrin) (99%), CH_3_COOH (99%), CH_3_COONa (99%), CH_3_COOC_2_H_5_ (99%) and C_2_H_5_OH absolute (99.8%) were purchased from Merck, India. Methionine amino acid (99%) was supplied from Loba Chemie, India. Standard solutions of the gemini surfactants methionine and ninhydrin were prepared by dissolving accurate amounts in a buffer solution of the required pH. Potentiometric analyses were completed on a digital pH meter manufactured by Hyderabad, India.

### 2.2. Synthesis of three double-headed gemini surfactants (16-6-16, GS-6; 16-5-16, GS-5 and 16-4-16, GS-4)

The three double-headed gemini surfactants (16-6-16, GS-6; 16-5-16, GS-5; and 16-4-16, GS-4) in the present study were synthesized in the laboratory using the process in previous work [55,56]. Accurate volumes of *α*,ω-dibromohexane and N, N-dimethylcetylamine were mixed at the molar ratio 1 : 2.1 in a two-necked experimental vessel using ethanol as the solvent at 353 K for 48 hr. The progress of the synthesis was checked by thin-layer chromatography (TLC). On completion, the experimental vessel was cooled to room temperature. The solvent present in the vessel was removed under vacuum pressure. As a consequence, a white solid was obtained. This solid was washed many times with ethyl acetate to remove any impurities. Next, the solid was placed to dry for two days in a vacuum desiccator filled with P_2_O_5_, avoiding using any solvents. The yield obtained was 70% to 90%. Using ^1^H NMR and elemental analyses, the purity of the gemini surfactants (GS-6, GS-5 and GS-4) was determined (electronic supplementary material, figures S1–S3 and table S1, electronic supplementary material) and found to agree with the values reported earlier [55].

### 2.3. Kinetic procedure

The methionine, buffer and surfactant solution were mixed in a reaction flask at the reaction temperature, and each run began by rapidly pouring ninhydrin into the flask holding the mixed solution. In the present case, the kinetics of methionine with ninhydrin in CH_3_COOH–CH_3_COONa buffer solvent was monitored to observe the impact of the double-headed gemini surfactants. Experiments were performed by measuring the absorbance with UV-vis spectrophotometry at fixed time intervals. A purple product was observed. Other relevant information on the kinetic procedure used in this study has been published elsewhere [57–61]. The reaction rate computed in the study was an average of three measurements.

### 2.4. Electrical conductivity

The specific electrical conductivities of the pure double-headed gemini surfactants (GS-6, GS-5 and GS-4) and their solutions mixed with reactants ([ninhydrin] = 5 × 10^−3^ mol dm^−3^ and [methionine] = 3 × 10^−4^ mol dm^−3^) were measured with a conductivity meter containing cell constant 1.0 cm^−1^. The cell material was glass. These measurements were carried out in double-surface glass tubes on a Systronics 306. The cell was calibrated with a KCl solution of suitable concentration, as suggested previously [62]. The study was performed at 303 K and 353 K; the temperature was maintained with a thermostated water/oil bath. The conductivities were recorded 2 min after each dilution, and at least three entries were taken as an average value. The CMC values were recorded in a conductivity versus different surfactant concentrations plot [63–67]. The CMC values measured in our study (H_2_O and H_2_O + ninhydrin + methionine) are shown below.

(a) 10^3^ [GS-6] (mol dm^−3^): 0.043, 0.032 at 303 K; 0.058, 0.050 at 353 K
(b) 10^3^ [GS-5] (mol dm^−3^): 0.034, 0.029 at 303 K; 0.055, 0.038 at 353 K
(c) 10^3^ [GS-4] (mol dm^−3^): 0.032, 0.025 at 303 K; 0.043, 0.029 at 353 K

### 2.5. Spectra formed between methionine and ninhydrin

**Figure 1.**
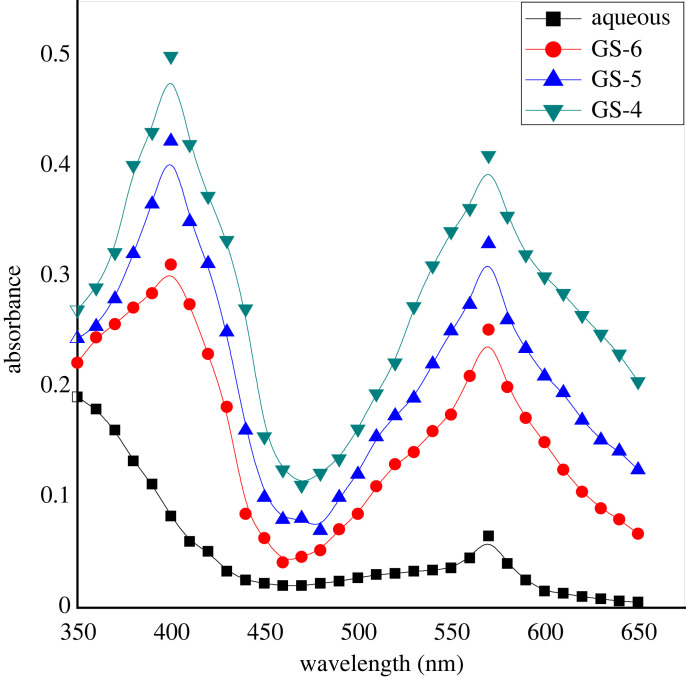
Several UV-vis spectra of methionine and ninhydrin presented in the absence and presence of three double-headed gemini surfactants at different wavelengths ranging from 350 nm to 600 nm at 353 K. [ninhydrin] = 5 × 10^−3^ mol dm^−3^, [methionine] = 3 × 10^−4^ mol dm^−3^, [gemini] = 30 × 10^−5^ mol dm^−3^ and pH 5.0.

Several UV-vis spectra of methionine and ninhydrin in water are presented with those of three double-headed geminis in figure 1. The absorbance was estimated at different wavelengths ranging from 350 to 600 nm at 353 K and pH 5.0. Figure 1 suggests that the absorbance is greater in surfactants than in aqueous and increases with the amount of gemini surfactants without changing the absorption maximum (*λ*_max_ = 570 nm). Therefore, it can be established that the product is identical in both media.

## 3. Results and discussion

### 3.1. Impact of pH on k_ψ_

**Figure 2.**
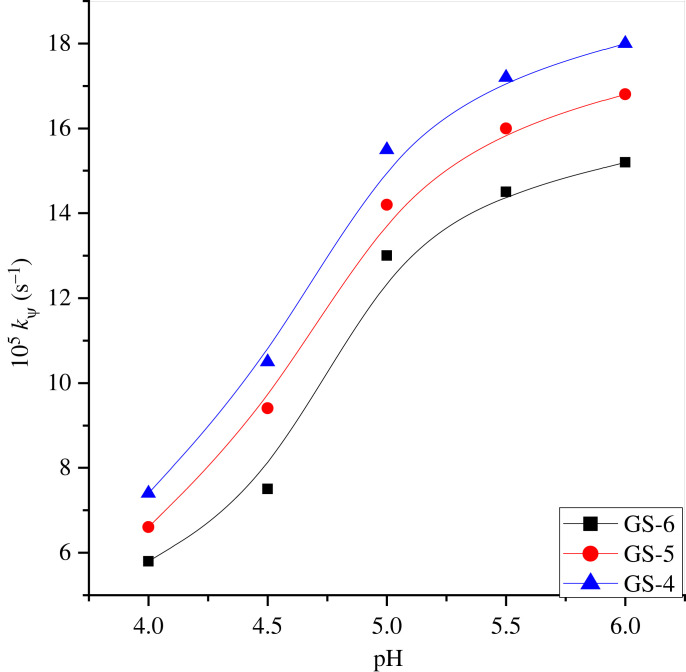
Impact of pH on *k*_ψ_ on ninhydrin–methionine reaction in double-headed gemini surfactants at 353 K. [ninhydrin] = 5 × 10^−3^ mol dm^−3^, [methionine] = 3 × 10^−4^ mol dm^−3^ and [gemini] = 30 × 10^−5^ mol dm^−3^.

The pH values have a significant impact on the ninhydrin–methionine reaction. The impact of varying the pH (4 to 6) was observed in the interaction of methionine with ninhydrin in the gemini micellar system at fixed temperatures and concentrations of amino acid and ninhydrin (electronic supplementary material, table S2). The plot of rate constant (*k*_ψ_) versus pH confirms that the *k*_ψ_-values increase rapidly at pH up to 5.0; after that, changes to the pH have almost no effect (figure 2). Since Schiff base development is an acid catalyzed and the optimum pH is 5.0 [68]. Our present product formed too has (>C = N-) type of linkage. Therefore, all the kinetics experiments were performed at pH 5.0.

### 3.2. Impact of methionine concentration on k_ψ_

Kinetic runs in the gemini micellar medium were performed at various methionine-fixing temperatures, with constant ninhydrin concentration and pH. The data obey the first-order rate law with respect to methionine concentration, as the rate constant values are independent of the initial concentrations of methionine. The *k*_ψ_ against several methionine concentrations are provided (table 1). Therefore, the rate equation can be written as equation (3.1).

3.1 $$\frac{d[\mathrm{product}]}{dt}=k_{\psi }\times [\mathrm{methionine}].$$

**Table 1.** Impact of methionine and temperature on *k_Ψ_* between methionine and ninhydrin reaction in double-headed geminis (30 × 10^−5^ mol dm^−3^) at ninhydrin (5 × 10^−3^ mol dm^−3^) and pH (5.0).

10^4^[methionine] (mol dm^−3^)	Temp. (K)	10^5^ *k*_ψ_ (s^−1^)		
		16-6-16	16-5	16 16-4-16
2.5	353	13.1	14.2	15.5
3.0	353	13.0	14.2	15.5
3.5	353	13.2	14.2	15.4
4.0	353	13.0	14.0	15.3
4.5	353	13.0	14.1	15.5
3.0	343	no reaction	no reaction	no reaction
3.0	348	11.5	12.5	14.0
3.0	353	13.0	14.2	15.5
3.0	358	16.1	17.2	18.5
3.0	363	20.0	21.5	23.0
3.0	368	25.5	26.8	28.4

### 3.3. Impact of ninhydrin concentration on *k_ψ_*

**Figure 3.**
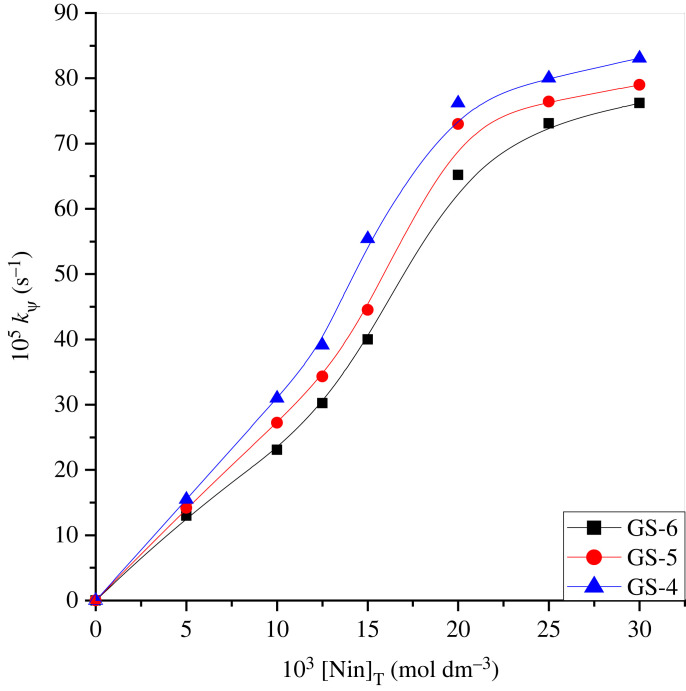
Impact of ninhydrin concentration on *k_ψ_* between ninhydrin–methionine reaction in double-headed gemini micellar system at 353 K. [methionine] = 3 × 10^−4^ mol dm^−3^, [gemini] = 30 × 10^−5^ mol dm^−3^ and pH 5.0.

The rate constants (*k*_ψ_) were estimated in geminis at various concentrations of ninhydrin by setting the rest of the reaction parameters as constant. The calculated rates corresponding to each ninhydrin concentration are reported in electronic supplementary material, table S2 (electronic supplementary material) and depicted graphically in figure 3. Figure 3 shows a nonlinear curve crossing through the origin; this indicates a fractional-order rate constant with respect to ninhydrin concentration.

### 3.4. Impact of temperature on *k*_ψ_

To examine the impact of temperature on the reaction rate of methionine with ninhydrin in buffer solution, kinetic analyses were performed at different temperatures: double-headed gemini surfactants were investigated from 343 K to 363 K. Meanwhile, other parameters, specifically the concentrations of ninhydrin and amino acid and pH, were fixed. The observed rate constants determined at varying temperatures are summarized in table 1. Using the data given in table 1. thermodynamic parameters (enthalpy of activation (Δ*H*^#^), entropy of activation (Δ*S*^#^) and energy of activation (*E*_a_)) were calculated with the linear least-squares regression method (table 2).

**Table 2.** Thermodynamic parameters (*E*_a_, *ΔH*^#^ and *ΔS*^#^), micellar rate constant (*k*_m_) and binding constants (*K*_C_ and *K*_D_) evaluated on study between methionine (3 × 10^−4^ mol dm^−3^) and ninhydrin (5 × 10^−3^ mol dm^−3^) reaction in double-headed surfactant medium.

	aqueous*^a^*	16-6-16*^b^*	16-5-16*^b^*	16-4-16*^b^*
*E*_a_ (kJ mol^−1^)	52.9	44.9	43.0	41.1
*ΔH*^#^ (kJ mol^−1^)	49.3	42.0	40.1	38.2
−Δ*S*^#^ (JK^−1^ mol^−1^)	184.0	190.2	191.7	194.0
10^3^ *k*_m_ (s^−1^)*^c^*	—	4.0	4.5	5.2
*K*_C_ (mol^−1^ dm^3^)*^c^*	—	76.0	74.0	72.0
*K*_D_ (mol^−1^ dm^3^)*^c^*	—	66.0	64.0	62.0

^a^The literature values (*E*_a_, Δ*H*^#^ and Δ*S*^#^) in aqueous are taken from [51].

^b^[gemini] = 30 × 10^−5^ mol dm^−3^ and

^c^at 353 K.

### 3.5. Impact of gemini on *k*_ψ_

**Figure 4.**
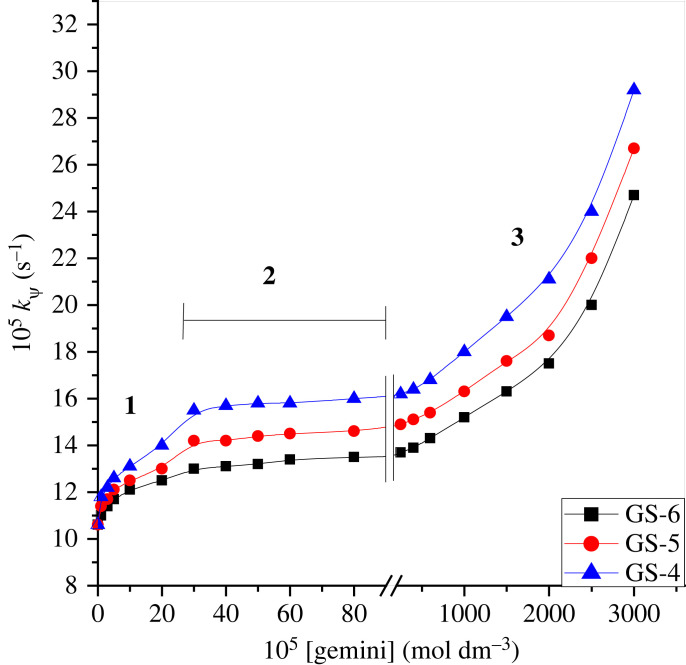
Impact of gemini concentration on *k*_ψ_ between ninhydrin–methionine reaction at 353 K. [ninhydrin] = 5 × 10^−3^ mol dm^−3^, [methionine] = 3 × 10^−4^ mol dm^−3^ and pH 5.0.

The impact of varying the quantity of gemini surfactant on the interaction of methionine with ninhydrin was studied at 353 K at constant concentrations of ninhydrin and methionine and fixed pH. At varied quantities of geminis, the *k*_ψ_-values were determined. These outcomes are presented in electronic supplementary material, table S3 (electronic supplementary material). The *k*_ψ_-values determined as a function of gemini quantity are plotted in figure 4. The data included in electronic supplementary material, table S3 confirm the first- and fractional-order kinetics in [methionine] and [ninhydrin] in geminis compared to that of the system without gemini surfactants; the same reaction mechanism is operating in both media.

## 4. Reaction mechanism

**Scheme 1.**
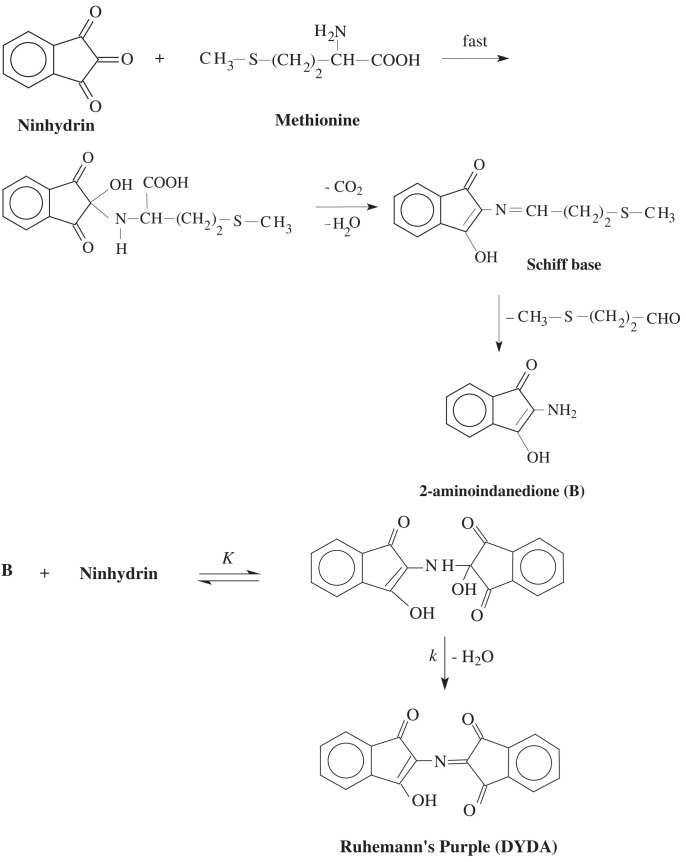
Mechanism of interaction of methionine amino acid with ninhydrin.

The mechanism of ninhydrin reactions is well established. The interaction mechanism of methionine with ninhydrin is shown in scheme 1. Condensation between the deprotonated amino group of methionine and the middle carbonyl group of ninhydrin follows. The reaction products generated from the interaction are carbon dioxide, Ruhemann's purple (DYDA) and aldehyde. However, the products generated depend upon several experimental reaction components: temperature, pH, and reactants. The reaction mechanism in this study has two steps. In the first step, a Schiff base intermediate comprising a double bonded N atom forms after decarboxylation. This intermediate is not stable and generates 2-aminoindanedione (B) upon hydrolysis. The second step reacts B with a second molecule of ninhydrin and produces Ruhemann's purple (DYDA) as the final purple-coloured product [68–72].

## 5. Quantitative impact of enhanced rate constant on the study in double-headed gemini (16-***s***-16) surfactants

**Scheme 2.**
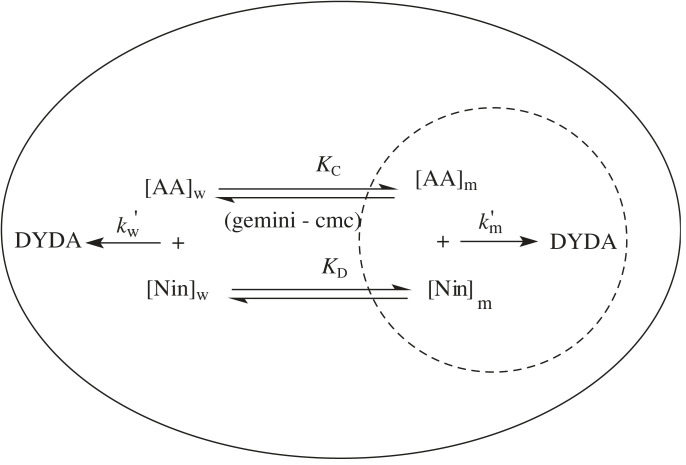
Interaction of methionine with ninhydrin in water and gemini system.

Under the current experimental conditions, the quantitative impact of enhanced *k*_ψ_ on methionine and ninhydrin at various surfactants may be analysed in terms of the micellar pseudo-phase model suggested by Menger & Portnoy [73] and used by Bunton [74,75] (scheme 2).

Here, [AA], m and w are the concentrations of methionine, surfactant system and water, respectively. *K*_C_, *K*_D_ and [Nin]_T_ are the binding constant for methionine and binding constant for ninhydrin and the total concentration of ninhydrin, respectively.

Considering scheme 2 provides equation (5.1):

5.1 $$k_{\psi }=\frac{{k^{\prime }}_{w}+{k^{\prime }}_{m}K_{C}([\mathrm{gemini}]\;-\mathrm{cmc})}{1+K_{C}(\;[\;\mathrm{gemini}]\;-\mathrm{cmc})\;},$$

where first-order rate constants ($k_{w}^{\prime }$ and ) are correlated to second-order rate constants (*k*_w_ and *k*_m_) as shown in equation (5.2):

5.2 $$k_{w}^{\prime }=k_{w}{\left[\mathrm{Nin}\right]}_{w},\;\mathrm{and}\;k_{m}^{\prime }=\frac{k_{m}{\left[\mathrm{Nin}\right]}_{m}}{\left([\mathrm{gemini}]-\mathrm{cmc}\right)}=k_{m}M_{N}^{S},$$

$M_{N}^{S}$ refers to ninhydrin concentration in the molar ratio of the Stern layer.

Combining equation (5.2) with the mass balance equation and substituting it into equation (5.1) produces equation (5.3):

5.3 $$k_{\Psi }=\frac{k_{w}{[\;\mathrm{Nin}]\;}_{T}+(K_{C}k_{m}-k_{w})M_{N}^{S}(\;[\;\mathrm{gemini}]\;-\mathrm{cmc})\;}{1+K_{C}(\;[\;\mathrm{gemini}]\;-\mathrm{cmc})\;}$$

$M_{N}^{S}$ was obtained from scheme 2 and the mass balance equation with [Nin]_T_ [76].

In order to evaluate *K*_C_ and *k*_m_, the CMC values were determined from the conductivity measurements. The best-fit KC and km were estimated by utilizing a nonlinear least-squares regression procedure, with *K*_D_ as an adjustable quantity (table 2). *K*_C_ and *k*_m_ were used in equation (5.3) to obtain the calculated rate constant values (*k*_ψcal_), which are presented in electronic supplementary material, table S3. The excellent agreement of the data (*k*_ψ_ and *k*_ψcal_) in electronic supplementary material, table S3 supports the proposed reaction mechanism.

**Figure 5.**
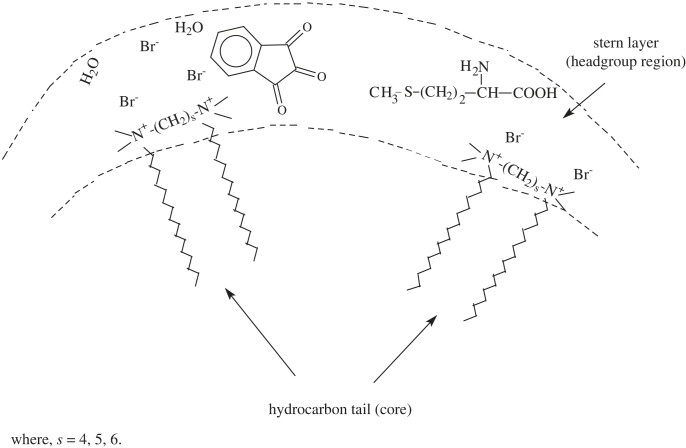
Micellar structure and plausible site of methionine and ninhydrin in double-headed gemini micellar system.

A typical graph of *k*_ψ_ and the concentration of the gemini surfactants is obtained. Due to considerably smaller CMC and the outstanding presentation of gemini surfactants on several fronts, their impact on the interaction of methionine with ninhydrin below and above these CMC values is studied. Diverse reactions are predicted to occur in a small zone (the Stern layer) of an ionic micelle (figure 5). A complete account of the impact of gemini surfactants on the rate constant is given below.

In part I of figure 4, the concentrations of the geminis are lower compared to the CMC. The rate constants in part I should not vary. However, figure 4 shows that the rates increase. This effect in rate constants may cause pre-micelles to form between the geminis and the reacting species. The existence of pre-micelles and the catalytic impact between reactants and surfactants has previously been reported at surfactant concentrations lower than the CMC [77,78].

However, no reaction is found; there is no variation in the *k*_ψ_-values in part II of figure 4 at gemini concentrations up to 400 × 10^−5^ mol dm^−3^. The effect of gemini concentration on *k*_ψ_ can only be obtained when a substrate is linked to the structure of the unmoving micelles [79,80]. The behaviour of parts I and II in figure 4 are identical to the results acquired from traditional surfactants [81,82]. Therefore, gemini surfactant systems greatly catalyze the studied system compared to traditional systems. Additionally, introducing double-headed geminis has advantageous effects in our study.

Considering part III of figure 4, the effects are very attractive in the concentration range from 400 to 3000 × 10^−5^ mol dm^−3^. Instead of fixed rate constants, a sudden fast increment in *k*_ψ_ was observed at higher gemini concentrations after the leveling-off region. This sudden change in rate possibly results from modifications to the micellar structure. These modifications of micellar structure at higher gemini concentrations are supported by the ^1^H NMR spectral data shown earlier [55,83]. Modifications in micellar morphologies and aggregates offer different reaction microenvironment conditions (less polar); in light of these conditions, at higher gemini concentrations, the rates increase greatly.

## 6. Thermodynamic parameters

Under the current kinetic conditions, several relevant thermodynamic quantities, that is, enthalpy of activation (Δ*H*^#^), entropy of activation (Δ*S*^#^), and energy of activation (*E*_a_), can be evaluated by varying the temperature from 343 K to 363 K in the presence of gemini micellar medium. The thermodynamic quantities determined in this way are listed in table 2. Comparing the data achieved in the 16-*s*-16 surfactant system with the data acquired in a pure water system, we note a low positive value of Δ*H*^#^ paired with a high value of Δ*S*^#^ in the surfactant system. This reduction in thermodynamic quantities in gemini surfactants is a consequence of stabilizing the transition state formed through the intermediate complex and adsorption of reactants onto the micellar surface of the Stern layer.

## 7. Conclusion

To determine the roles of several components, their influence on the interaction of methionine with ninhydrin in buffer solution was studied using UV-vis spectroscopy. Their different effects on the reaction are summarized. The impact of different concentrations of three double-headed gemini surfactants with varying spacer lengths (gemini concentrations varied from 0 to 3000 × 10^−5^ mol dm^−3^) on methionine and ninhydrin was measured. The CMC values of the micellar system with and without reactants were determined using the conductometric technique. The CMC is a useful quantity for understanding the ability of surfactants to stimulate different organic reactions.

Electronic supplementary material, table S3 (electronic supplementary material) confirms that compared to pure water, the gemini micellar medium is outstanding for accelerating and catalyzing at gemini concentrations lower than their CMC values. Catalytic behaviour in micelles occurs because organic reactants are incorporated into the micelles, where the reaction occurs in the small zone of the micellar Stern layer. Owing to this unusual property of gemini surfactants, that is, the lower amounts of surfactant needed for the reaction, this study used significantly smaller amounts of the reactants in addition to maximizing the reaction rate. Consequently, the applicability of the system and method improves. Thus, the gemini surfactants used in this study are more economical and environmentally suitable. These features allow them to be categorized as green surfactants. However, the present study of gemini micellar media may stimulate and provide new tactics for ninhydrin reactions with amino groups and find improved applications in forensic work related to the visualization of ninhydrin-developed fingerprints.

## Data Availability

Data that support this study have been uploaded as electronic supplementary material [84].
